# An experimental study to inform adoption of mindfulness-based stress reduction in chronic low back pain

**DOI:** 10.1186/s43058-022-00335-w

**Published:** 2022-08-06

**Authors:** Salene M. W. Jones, Karen J. Sherman, Zoe Bermet, Lorella G. Palazzo, Cara C. Lewis

**Affiliations:** 1 Fred Hutchinson Cancer Research Center, 1100 Fairview Ave N, Seattle, WA 98109 USA; 2 Kaiser Permanente Washington Health Research Institute, Seattle, USA

**Keywords:** Mindfulness, Low back pain, Mediation

## Abstract

**Background:**

Chronic low back pain is a common and sometimes disabling condition, and mindfulness-based stress reduction is recommended as a first line of therapy. This study tested whether different descriptions of mindfulness training altered based on influential intervention characteristics increased adoption intentions.

**Methods:**

People with chronic low back pain (*n* = 452) were randomized to review one of four mindfulness training descriptions in an online survey using a 2 × 2 factorial design. The first factor was evidence strength and quality with relative advantage (ER). The second factor was adaptability, trialability, complexity, and design quality and packaging (AD). Each factor had two levels: a description of standardized mindfulness training that described each intervention characteristic and a patient-centered description emphasizing flexibility and patient testimonials. The primary outcomes were intentions to try mindfulness training and practice mindfulness at home. Using structural equation modeling with a bootstrapped distribution, we tested six mediators, three of which are Theory of Planned Behavior predictors of intention—self-efficacy, norms, and attitudes— and the other three are predictors of adoption—feasibility, appropriateness, and acceptability.

**Results:**

Overall, the mindfulness training descriptions were not associated with an increase in intentions compared to the classic vignette (11/12 *p*’s > 0.05). Most descriptions were unrelated to mediators except the classic ER with patient-centered AD was associated with higher self-efficacy/control and feasibility (*p*’s ≤ 0.05; standardized effect range: 0.111–0.125). Self-efficacy/control (training standardized coefficient: 0.531, home: 0.686), norms (training: 0.303, home: 0.256), and attitudes (training: 0.316, home: 0.293) were all positively associated with intentions to adopt mindfulness training and home practice. Feasibility (training: 0.185; home: 0.293) and acceptability (training: 0.639; home: 0.554) were positively related to intentions to adopt mindfulness training. Appropriateness was related to intentions to adopt home practice (0.187) but not mindfulness training (0.100). None of the indirect effects from experimental group to intentions was significant (all *p*’s > 0.05).

**Conclusions:**

Self-efficacy/control and acceptability may be key mediators for increasing patient adoption of mindfulness. Because experimental manipulation did not substantially change intentions to adopt mindfulness, the presentation and delivery of MBSR may need to be tailored to the individual patient’s needs rather than a specific format for chronic low back pain.

**Supplementary Information:**

The online version contains supplementary material available at 10.1186/s43058-022-00335-w.

Contributions to the literature

- An adaptable mindfulness training program could increase feasibility and self-efficacy.
- Increasing patient feelings of acceptability towards mindfulness could improve adoption.
- Presentation of mindfulness training may need to be tailored to each individual patient.

## Background

Chronic low back pain is common, with an estimated 40% of American adults experiencing persistent low back pain at some point in their lives [1]. Chronic low back pain is defined by pain in the lower back that persists at least 12 weeks beyond the first occurrence of the back pain [2]. Around 6 in 10 people with chronic low back pain report moderate to severe pain or disability [3]. Treating chronic low back pain is particularly challenging because the underlying damage or disease is often not found by medical investigation [4].

Both the Centers for Disease Control and Prevention and the American College of Physicians recommend non-pharmacologic treatments for chronic low back pain [5, 6]. One of these treatments is mindfulness-based stress reduction (MBSR [7];). MBSR teaches participants how to use mindfulness, which is a purposeful way of focusing on the present moment, nonjudgmentally, and focusing on one stimulus. Developed by Jon Kabat-Zinn in the 1970s and adapted from the Buddhist practice of mindfulness meditation, a typical MBSR treatment involves 28 h of classes and 31 h of home practice over 8 weeks teaching multiple mindfulness techniques such as walking meditation and breathing meditation. Clinical trials have shown that MBSR is effective for chronic low back pain [8–11]. However, MBSR requires a large time commitment and is not easy to learn. Finding ways to adapt implementation of MBSR to increase patient adoption could help address the widespread suffering from chronic low back pain.

**Fig. 1:**
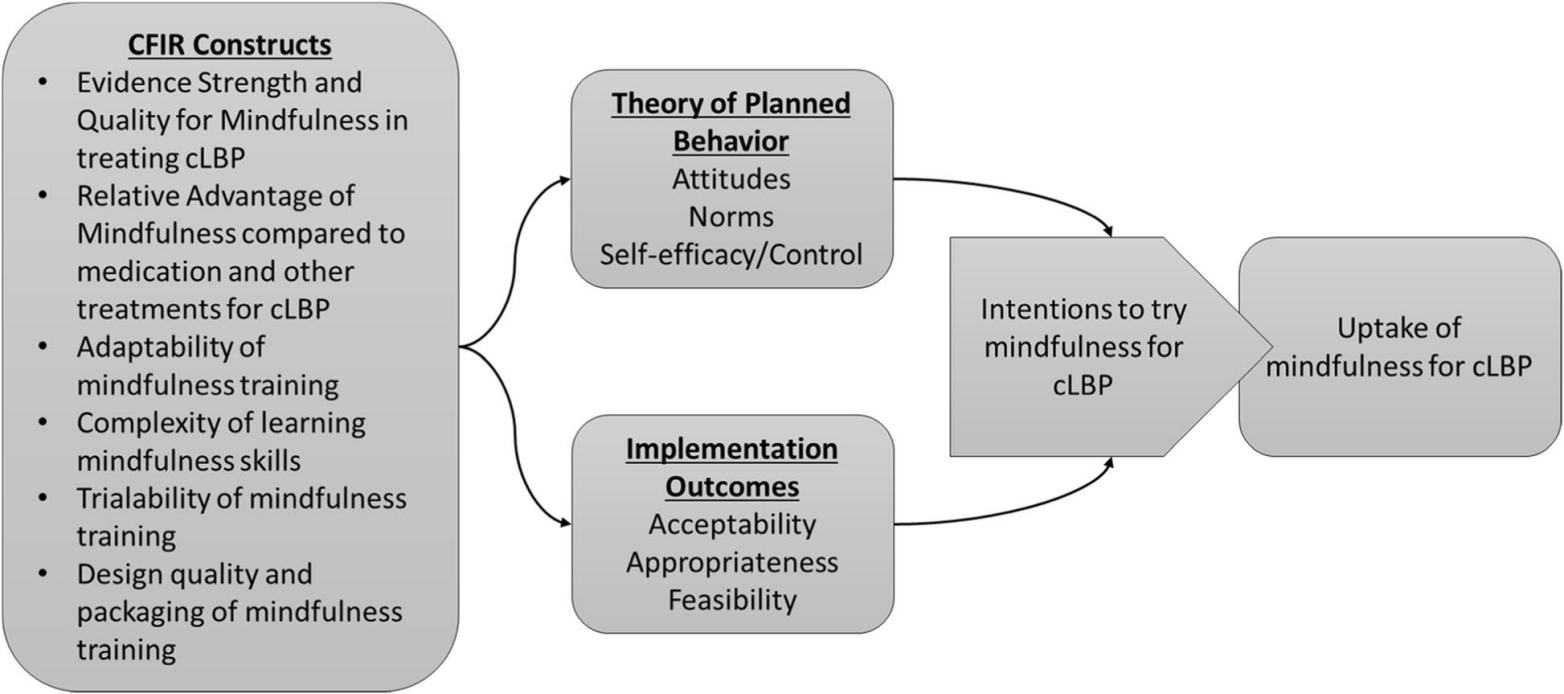
Theoretical model for the study. CFIR Consolidated Framework for Implementation Research, cLBP chronic low back pain

To investigate ways to improve uptake or adoption of mindfulness training, we used the Consolidated Framework for Implementation Research (CFIR [12];), the Theory of Planned Behavior [13], and the framework on outcomes for implementation research (Fig. 1 [14];). CFIR delineates different barriers and facilitators of evidence-based practice adoption into five domains, including intervention characteristics. For this study, we focused on intervention characteristics as a first step to ensure direct-to-consumer dissemination of the intervention was optimized before tackling the complex work of implementation. Our aim was to ensure any adaptations of mindfulness training would make the intervention most attractive to potential participants. The evidence-based, influential intervention characteristics in CFIR, which builds from and incorporates parts from Rogers’ Theory of Diffusion [15], include evidence strength and quality, relative advantage, adaptability, trialability, complexity, and design quality and packaging. Evidence strength and quality refers to perceptions of the research and other evidence supporting the intervention, mindfulness training in this case. Relative advantage is defined as benefits and drawbacks of the intervention compared to some alternative. Adaptability refers to whether the intervention can be tailored to individual needs. Trialability is whether the intervention can be tried on a small scale or reversed once started. Complexity is the difficulty level of implementing or using the intervention. Design quality and packaging refers to how the intervention is presented or put together. These six intervention characteristics informed the experimental groups for the current study.

The Theory of Planned Behavior [13, 16] outlines several cognitive factors that influence whether a person adopts a health behavior such as mindfulness for chronic low back pain. The first cognitive construct is attitudes or how a person feels about a health behavior or their personal judgments about adopting the behavior. Norms refer to the person’s perceptions of what others think of the health behavior. Self-efficacy and perceived control are two related constructs, often grouped together, and refer to perceptions of whether the person can complete the health behavior and whether they have control over engaging in the behavior. Numerous studies have shown that these three constructs are strongly associated with intentions to engage in health behaviors and actual health behavior [17].

The third framework informing our study was Proctor’s framework of implementation outcomes [14]. When an evidence-based practice is implemented, the success of that implementation can be measured using these outcomes. For this study, we focused on the acceptability, appropriateness, and feasibility as these are predictors of adoption. Acceptability refers to whether a patient personally feels positive towards the intervention. Appropriateness encompasses the patient’s beliefs about whether the intervention fits for their condition and for people like them. Feasibility is defined as whether the intervention can actually be used by the patient.

The current study was a randomized experiment that investigated the effect of mindfulness training descriptions on intentions to try mindfulness classes and home practice and to inform implementation of mindfulness for chronic low back pain. We used intervention characteristics as our independent variable and varied the mindfulness training descriptions by evidence strength and quality, relative advantage, adaptability, trialability, complexity, and design quality and packaging. Our outcomes were intentions to try mindfulness training (classes) and mindfulness home practice. The Theory of Planned Behavior constructs (attitudes, norms, self-efficacy/control) and implementation outcomes (acceptability, appropriateness, feasibility) were conceptualized as mediators of the effect of intervention characteristics on intentions. Results from this study can inform dissemination strategies for improving the adoption of mindfulness training for chronic low back pain.

## Methods

### Experimental stimuli

Our experimental stimuli consisted of four brief written descriptions of MBSR. The descriptions varied based on the intervention characteristics and were informed by focus groups and cognitive interviews with people with chronic low back pain. During the focus groups and cognitive interviews, two key findings emerged. First, participants had difficulty both distinguishing evidence strength and quality from relative advantage and distinguishing adaptability, trialability, complexity, and design quality and packaging. For this reason, we combined evidence strength and quality with relative advantage (ER) in the MBSR descriptions and combined adaptability, trialability, and complexity with design quality and packaging (AD). Each description started with text on the ER characteristics of mindfulness training and ended with text on the AD characteristics of mindfulness training. Each set of intervention characteristics (ER, AD) had two levels. The first level, referred to as classic, described the traditional mindfulness training as developed by Jon Kabat-Zinn. The second level, referred to as patient-centered, described mindfulness training in more flexible and personalized terms. The classic ER description consisted of summaries of scientific evidence, recommendations by professional medical associations, and comparisons to medications for chronic low back pain. The patient-centered ER description used patient testimonials and quotes to support the use of mindfulness training for chronic low back pain. The classic AD condition described the regimented structure of traditional mindfulness training (2-h classes every week; need for home practice all the other days; need to learn each mindfulness technique in a set order; mindfulness described as skill that needs to be learned). The patient-centered AD description portrayed mindfulness training as having a flexible class structure and modality (in-person, online) and ability to choose which techniques worked for each individual and portrayed mindfulness as a natural way of being that was easily accessible. The study team drafted each description based on focus group results and the descriptions were then reviewed by participants in the cognitive interviews. A plain language communication specialist also revised the descriptions to ensure the text was at an 8th grade reading level or lower (see Additional files 1, 2, 3 and 4 for descriptions).

### Experimental groups

The study was a 2 × 2 randomized experiment. The intervention characteristics defined both factors: ER and AD. Each factor had two levels: classic and patient-centered descriptions of mindfulness training. The first factor had participants randomized to classic or patient-centered ER. The second factor had participants randomized to classic or patient-centered AD. The four groups were classic ER/classic AD (described as classic only subsequently), classic ER/patient-centered AD, patient-centered ER-classic AD, and patient-centered ER/patient-centered AD (described as patient only subsequently). An additional aim of the study, reported in other publications, was to assess demographic and disease factors associated with intentions to try classic mindfulness training, and all participants reviewed the classic only description. To prevent order effects, participants randomized to review one of the patient-centered descriptions were also randomized by whether they reviewed the classic only description first or after a more patient-centered version (see Table 1).

**Table 1:** Experimental groups. In analyses, groups 2 and 3 were combined, groups 4 and 5 were combined, and groups 6 and 7 were combined

Experimental group	Order of stimuli	Abbreviations for four experimental groups
1	Classic ER/classic AD only	All classic
2	Classic ER/classic AD then classic ER/patient-centered AD	Classic ER/patient AD
3	Classic ER/patient-centered AD then classic ER/classic AD	Classic ER/patient AD
4	Classic ER/classic AD then patient-centered ER-classic AD	Patient ER/classic AD
5	Patient-centered ER-classic AD then classic ER/classic AD	Patient ER/classic AD
6	Classic ER/classic AD then patient-centered ER/patient-centered AD	All patients
7	Patient-centered ER/patient-centered AD then classic ER/classic AD	All patients

*ER* evidence strength and quality, relative advantage, *AD* adaptability, trialability, complexity, and design quality and packaging

The classic ER text focused on scientific studies supporting mindfulness and professional physician organizations recommending mindfulness. The classic ER text included phrases such as “Mindfulness is a scientifically proven treatment for chronic low back pain” and “But more importantly, they [mindfulness benefits] are also similar to the benefits people experience from most pain medications—except without the side effects.” The patient-centered ER text used patient testimonials to explain the benefits of mindfulness for everyday function. The patient-centered ER text included phrases such as “People around the world have used mindfulness to help with chronic low back pain because they experience positive results” and “Here are some comments from people who have used mindfulness to improve their pain or make it less disruptive in their lives.” The classic AD text emphasized the rigorous, almost strict protocol of the standard mindfulness training. The classic AD text included phrases such as “Classes are held once a week for 8 weeks. Each class lasts about 2 to 2.5 hours” and “Because mindfulness is a new experience for many people, the best way to learn it is by doing a mindfulness training course.” The patient-centered AD text focused on adapting mindfulness training to each participant and providing options rather than a predetermined curriculum. The patient-centered AD text included phrases such as “Mindfulness is a natural way of paying attention” and “You can try each technique and continue using those you find helpful.”

### Participants and procedures

Participants were recruited through Kaiser Permanente Washington Health Research Institute (KPWHRI) between December 2019 and August 2020. Eligibility criteria included chronic low back pain for at least 6 months, age 18 or older, at least a 3 out of 10 pain interference or pain intensity, not currently pregnant, not currently in any legal cases related to the back pain, able to read English, and able to provide informed consent. Potentially eligible participants were identified through the Kaiser Permanente Washington (KPWA) electronic health record based on whether they had two medical visits for back pain during the prior 12 months, though it was impossible to know how many would have met the criteria for chronic low back pain. Potentially eligible participants were mailed a letter describing the study and providing a link to the screening survey. Potential participants completed a Web-based screening survey that assessed whether they met eligibility criteria. Eligible persons were then sent to the online consent form, and if they consented, they then completed the survey online. Potential survey participants were randomized to one of the seven groups before mailing the letters with group 1 weighted at twice the number of participants as the other six groups (see Table 1). Letters were mailed in 15 waves to potentially eligible KPWA members. Across the 15 waves, 733 people completed at least one item in the screening survey, 710 completed enough questions to determine eligibility, and 457 screened as eligible and completed the survey. The survey consisted of reading one or two of the mindfulness training descriptions, depending on the experimental group, and then answering questions about intentions, attitudes, and reactions to mindfulness training for each of the descriptions. The KPWA institutional review board approved all procedures.

### Measures

The Theory of Planned Behavior measures were developed specifically for this project using an established manual that recommends surfacing content/language via focus groups and cognitive interviews [16]. We conducted three focus groups to identify content around Theory of Planned Behavior constructs, developed a questionnaire using the participants own words, and then conducted cognitive interviews to ensure the questionnaire was understandable. One item assessed intentions to try mindfulness classes. Three items assessed intentions to practice mindfulness at home (Cronbach’s alpha for this sample was 0.959). The intention items were rated on a 7-point Likert scale. Attitudes were assessed using four items, each asking participants to rate mindfulness on a bipolar scale (harmful-beneficial, good-bad, worthless-useful, necessary-unnecessary; Cronbach’s alpha = 0.878). Perceived norms were assessed using two items, both rated on a 7-point Likert scale (Cronbach’s alpha = 0.951). Self-efficacy was assessed using three items and perceived control was also assessed with three items (Cronbach’s alpha = 0.784). The self-efficacy and control items were assessed using the same 7-point Likert scale as the other items. Implementation science outcomes of acceptability (3 items), appropriateness (2 items), and feasibility (3 items) were assessed using measures previously developed and shown to be reliable and valid [18]. Acceptability is how palatable the participant perceives mindfulness training to be for themselves (Cronbach’s alpha = 0.925). Appropriateness is how compatible the participant perceives mindfulness training to be for chronic low back pain (Cronbach’s alpha = 0.924). Feasibility is how successfully the participant believes mindfulness training could be used for chronic low back pain (Cronbach’s alpha = 0.820). The implementation outcome measures use a 5-point Likert scale ranging from strongly disagree (1 point) to strongly agree (5 points) and all items are phrased positively.

The survey also asked questions about demographics and back pain. Time since back pain started, pain interference on a 0 to 10 scale, and previous use of back treatments were assessed. For back pain treatments, participants were provided with a list of the following treatments and asked if they had used these: opioids, injections, exercise, psychological counseling, mindfulness therapy, yoga, and other treatments. Participants also completed Patient-Reported Outcome Measurement Information System 4-item assessments for pain interference, sleep disturbance, and physical function [19–21].

### Statistical analyses

Preliminary analyses consisted of comparison of characteristics across the experimental groups and a series of multiple analysis of variance (MANOVA) models. The demographic and disease characteristics of the experimental groups were compared using bivariate tests (*t*-tests, chi-squares). The factors for the MANOVAs were the two experimental groups (classic ER vs. patient-centered ER; classic AD vs. patient-centered AD). Two MANOVAs were run, one for the Theory of Planned Behavior constructs and one for the implementation science outcomes. After the MANOVAs, we ran a series of mediation analyses using structural equation modeling and a bootstrapped distribution. The predictors in the mediation analyses were the experimental groups with the classic only group as the reference group. The mediators were either the Theory of Planned Behavior constructs or the implementation science outcomes. The outcomes were the intention item for mindfulness classes or the three items for intention to try mindfulness home practice. All mediators and outcomes with multiple items were modeled as latent variables. A total of four mediation models were run: Theory of Planned Behavior with intention for mindfulness classes, Theory of Planned Behavior with intention for mindfulness home practice, implementation outcomes with intention for mindfulness classes, and implementation outcomes with intention for mindfulness home practice. Standardized coefficients were used to describe the relationships in the model. Mediation models controlled for age and gender. To be included, participants had to have complete data on all variables used in the analyses. MANOVAs and mediation analyses were run with SPSS and AMOS version 27.

## Results

### Sample description

A total of 457 persons with chronic low back pain consented to participate and completed some of the questionnaire. Due to missing data, 5 persons were excluded for a final sample of 452. The following number of participants were randomized to each intervention group: 97 in classic only, 113 in classic ER-patient-centered AD, 116 in patient-centered ER-classic AD, and 126 in patient-centered only. On the ER factor, the two groups did not differ on physical function, sleep problems, age, gender, ethnicity, education level, and back pain treatment (*p*’s > 0.05). ER groups did differ on pain interference (*p* = 0.042; 0.95 points with 4.99 standard deviation; Cohen’s *d* = 0.191). On the AD factor, the two groups did not differ on pain interference, physical function, sleep problems, gender, ethnicity, education level, and back pain treatment (*p*’s > 0.05). The AD factor did differ by age (*p* = 0.014; 3.74 years with 16.23 standard deviation; Cohen’s *d* = 0.231).

The participant demographics were consistent with previous studies of chronic low back pain and the KPWA population (Table 2). Participants were on average middle aged (mean age 53.22), female (67.0%), and white (84.3%). Most were still working (50.7%) and had a bachelor’s degree or higher (59.8%). Mean pain interference in the PROMIS scale was 63.20, much higher than the population mean of 50.0. Mean PROMIS physical function was 34.02, much lower than the population mean of 50.0. Attitudes, norms, and self-efficacy/control were slightly positive (4.42 to 5.85 on a 1–7 scale). Implementation outcomes were positive based on the mean responses (3.83 to 3.95 on a 1–5 scale) being around the “slightly agree” response option.

**Table 2:** Sample description

Characteristic	Mean (SD) or *N* (%)
Age	53.22 (16.33)
Gender	
Male	142 (31.3)
Female	303 (67.0)
Others	7 (1.5)
Race/ethnicity	
White	381 (84.3)
Hispanic	17 (3.8)
Black	18 (4.0)
Asian	28 (6.2)
Native American	13 (2.9)
Pacific Islander	6 (1.3)
Employment	
Working	229 (50.7)
Not working, looking for work	7 (1.5)
Not working, not looking for work (sick leave, disability, student)	26 (5.7)
Retired	131 (29.0)
Temporarily laid off	8 (1.8)
Homemaker	7 (1.5)
Others	37 (8.2)
Education	
High school diploma or less	22 (4.9)
Some college	83 (18.4)
Associates or certificate	74 (16.3)
Bachelor’s degree	140 (31.0)
Graduate degree	130 (28.8)
Back pain interference (0–10 scale)	5.53 (1.71)
PROMIS Pain interference	63.20 (4.98)
PROMIS Physical function	34.02 (4.78)
PROMIS Sleep problems	54.07 (7.88)
Attitudes (1–7 scale)	5.65 (1.14)
Norms (1–7 scale)	4.42 (1.59)
Self-efficacy/control (1–7 scale)	5.85 (0.92)
Acceptability (1–5 scale)	3.95 (0.85)
Appropriateness (1–5 scale)	3.83 (0.92)
Feasibility (1–5 scale)	3.88 (0.67)
Intentions for MBSR classes (1–7 scale)	4.98 (1.65)
Intentions for MBSR home practice (1–7 scale)	5.41 (1.48)

Norms, self-efficacy/control, acceptability, appropriateness, feasibility, and intentions all used a Likert scale with 1 corresponding to strongly disagree and the highest value corresponding to strongly agree

### Theory of planned behavior

The MANOVAs for the Theory of Planned Behavior constructs suggested an overall effect for the AD factor (Hotelling’s trace = 0.024, *F*(3, 446) = 3.611, *p* = 0.013) but not for the ER factor (Hotelling’s trace = 0.014, *F*(3, 446) = 2.065, *p* = 0.104) or the interaction of ER and AD (Hotelling’s trace = 0.012, *F*(3, 446) = 1.829, *p* = 0.141). The tests for individual outcomes in the AD factor showed a significant effect for self-efficacy/control (*F*(1, 448) = 9.214, *p* = 0.003) wherein participants who read the patient-centered AD description reported more self-efficacy and perceived control than participants reading the classic AD description. The tests for the effects of AD on attitudes (*F*(1, 448) = 0.579, *p* = 0.447) and norms (*F*(1, 448) = 0.081, *p* = 0.777) were not significant.

**Fig. 2:**
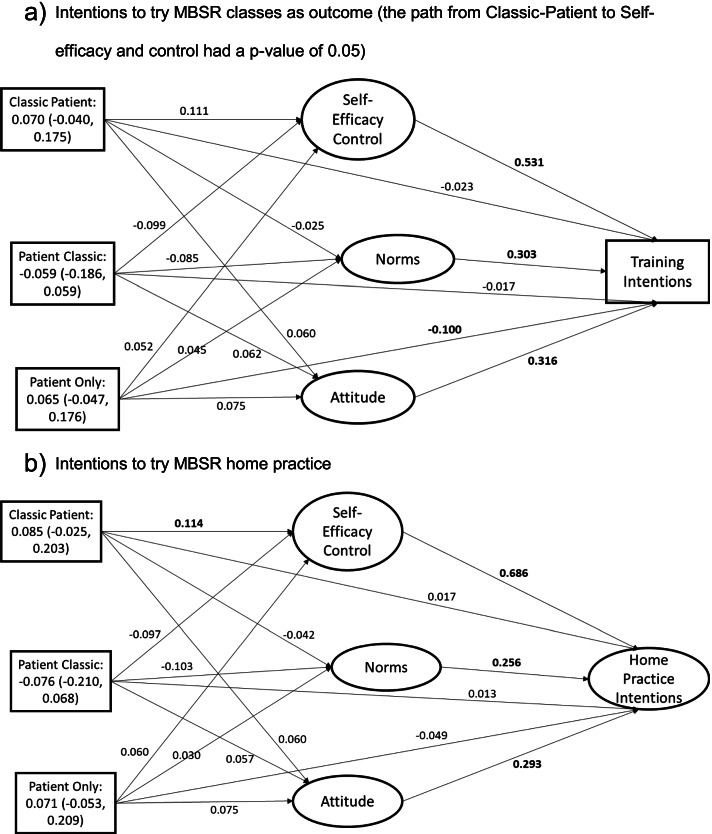
Mediation model for Theory of Planned Behavior mediators. Indirect effects for each experimental group are in the rectangle representing each group with the point estimate (95% confidence interval). Bold indicates significant at *p* < 0.05. **a** Intentions to try MBSR classes as outcome (the path from classic patient to self-efficacy and control had a *p*-value of 0.05). **b** Intentions to try MBSR home practice

The mediation models for the Theory of Planned Behavior constructs did not show experimental effects on intentions but did show that the mediators were associated with intentions (Fig. 2). For intentions to try the mindfulness classes (Fig. 2a), the indirect effect for the classic ER-patient-centered AD was not significant (0.070, 95% CI: −0.040, 0.175). Indirect effects on intentions to try mindfulness classes were also non-significant for patient-centered ER-classic AD (−0.059, 95% CI: −0.186, 0.059) and patient only (0.065, 95% CI: −0.047, 0.176). Only one direct group effect on intentions was significant, patient only (−0.100, *p* < 0.05), such that this group had lower intentions than the classic only group. The direct pathways from the Theory of Planned Behavior constructs to intentions were statistically significant (*p* < 0.05; standardized coefficient for self-efficacy/control = 0.531, attitudes = 0.316, norms = 0.303). Self-efficacy and control had the strongest association with intentions, such that a one standard deviation increase in self-efficacy/control was associated with a 0.531 standard deviation increase in intentions.

The mediation model for Theory of Planned Behavior mediators and intentions to try home practice resembled the model for intentions to try mindfulness classes (Fig. 2b). There was an experimental effect of classic ER-patient-centered AD on self-efficacy/control, but the effect was small (0.114, *p* < 0.05). All the confidence intervals for the indirect effects of the experimental intervention included zero, indicating no mediated effects of the experimental groups. None of the direct group effects on home practice intentions was significant (*p*’s > 0.05). The Theory of Planned Behavior constructs were significantly associated with home practice intentions (*p*’s < 0.05; standardized coefficients for self-efficacy/control: 0.686, attitudes: 0.293, norms: 0.256).

### Implementation outcomes

The MANOVA for the implementation outcomes showed an overall effect for the AD factor (Hotelling’s trace = 0.022, *F*(3,451) = 3.265, *p* = 0.021) but not the ER factor (Hotelling’s trace = 0.005, *F*(3, 451) = 0.726, *p* = 0.537) or the interaction of the ER and AD factors (Hotelling’s trace = 0.001, *F*(3, 451) = 0.192, *p* = 0.902). The outcome-specific between-subjects analyses for the AD factor showed a significant difference for feasibility (*F*(1, 453) = 6.489, *p* = 0.011) such that the patient-centered descriptions had more feasibility than the classic descriptions. The AD factor did not show significant differences for acceptability (*F*(1, 453) = 0.312, *p* = 0.577) nor appropriateness (*F*(1, 453) = 0.849, *p* = 0.357).

**Fig. 3:**
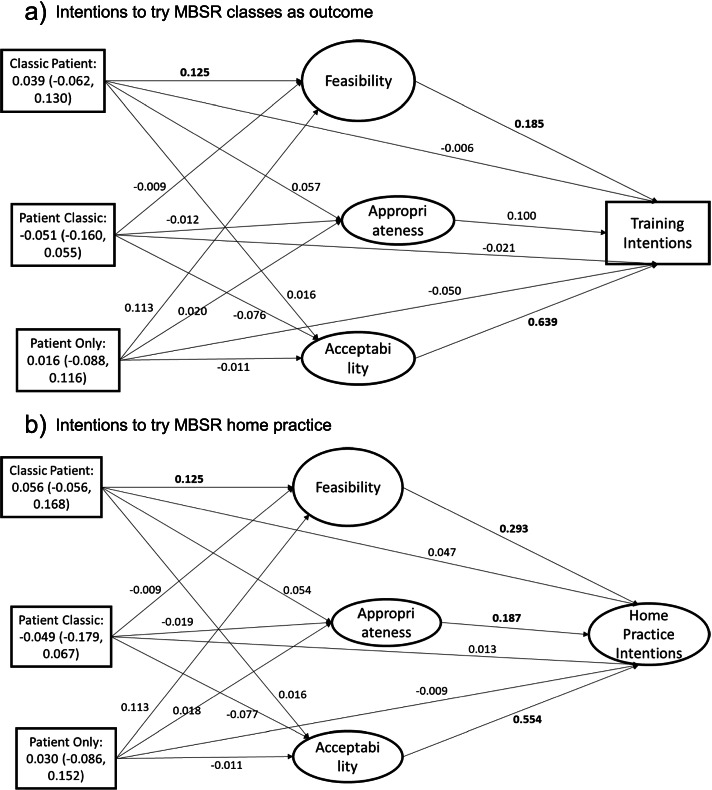
Mediation model for implementation outcome mediators. Indirect effects for each experimental group are in the rectangle representing each group with the point estimate (95% confidence interval). Bold indicates significant at *p* < 0.05. **a** Intentions to try MBSR classes as outcome. **b** Intentions to try MBSR home practice

The implementation outcome mediation models showed only one intervention effect on mediators but no direct effects of experimental group on intentions. For intentions to try MBSR training (Fig. 3), classic ER-patient-centered AD had a significant effect on feasibility (Fig. 3a, 0.125, *p* < 0.05) but no other direct intervention effects on other mediators (appropriateness, acceptability) or the outcome were significant (*p*’s > 0.05). The association of acceptability (standardized coefficient: 0.639, *p* < 0.05) and feasibility (standardized coefficient: 0.185, *p* < 0.05) with training intentions were significant but the association of appropriateness (standardized coefficient: 0.100, *p* > 0.05) was not significant. No indirect effects were significant for the experimental groups on training intentions.

For intentions to try home practice, results were similar to the mediation model for intentions to try mindfulness classes. The classic ER-patient-centered AD had a significant effect on feasibility (Fig. 3b, 0.125, *p* < 0.05) but not acceptability nor appropriateness (*p*’s > 0.05). The other two experimental groups did not differ from the reference group (classic only) for effects on implementation outcomes (feasibility, appropriateness, acceptability). The mediators were all associated with home practice intentions. Acceptability (standardized coefficient: 0.554, *p* < 0.05), feasibility (standardized coefficient: 0.293, *p* < 0.05), and appropriateness (standardized coefficient: 0.187, *p* < 0.05) were significantly associated with home practice intentions. All confidence intervals for the experimental groups’ indirect effects contained zero, indicating no significant indirect effects of the experimental groups on home practice intentions.

## Discussion

This study examined the effects of different mindfulness training descriptions, varied by intervention characteristics, on intentions to try mindfulness and whether Theory of Planned Behavior constructs and implementation outcomes mediated the relationships. Descriptions that were more patient centered in the adaptability, trialability, complexity, and design quality and packaging of mindfulness training increased self-efficacy, perceived control, and feasibility of trying mindfulness. In particular, the description with classic, scientific evidence and flexibility in class completion was associated with more self-efficacy, perceived control, and feasibility. None of the descriptions affected intentions to try mindfulness classes or home practice, either directly or indirectly through Theory of Planned Behavior constructs or implementation outcomes. All Theory of Planned Behavior constructs (self-efficacy/control, norms, attitudes) and nearly all implementation outcomes (feasibility, acceptability) were associated with higher intentions to try mindfulness classes. All implementation outcomes (feasibility, appropriateness, acceptability) and Theory of Planned Behavior constructs were associated with higher intentions to try mindfulness home practice.

The results for Theory of Planned Behavior and implementation outcomes showed that how the participant felt personally about mindfulness training influenced their intentions to try mindfulness for chronic low back pain. Self-efficacy was the strongest predictor of intentions in the Theory of Planned Behavior model as was acceptability in the implementation outcome model. Both constructs refer to how a participant feels personally that they can participate in mindfulness training and whether they feel mindfulness training could help their chronic low back pain. More general concepts, such as appropriateness, and constructs referring to others’ perceptions, such as norms, were less strongly related to intentions. These results suggest that tailoring mindfulness training to each individual patient, both in treatment structure and evidence for the treatment, may be needed to increase adoption.

An interesting finding from this study was that acceptability was the strongest predictor in the implementation outcome model but attitude was not as strong a predictor as self-efficacy in the Theory of Planned Behavior model. Another seemingly paradoxical finding was the strong relationship of self-efficacy to intentions but the comparatively weaker relationship of feasibility to intentions. While the constructs might seem similar, differences in item content could explain the contradictory results. Both self-efficacy and acceptability referred to how the participant felt about trying mindfulness training for themselves while attitudes and feasibility both assessed general perceptions such as whether mindfulness training was good or bad for chronic low back pain. This further reinforces the implication that whether a participant feels personally positive towards a treatment and whether they believe they themselves can engage in the treatment is more crucial for adoption and improving implementation than general beliefs. The need to personalize messaging and delivery of mindfulness training further explains our lack of effects for the experimental groups. Participants were randomized to mindfulness training descriptions and the stimulus was not tailored to what was most important to the participant. Some participants may need greater structure while others need flexibility and some may need prescriptions for specific techniques while others prefer to choose the techniques for themselves. Overall, our results suggest having options for mindfulness training participation and how mindfulness training is delivered may be the best strategies when implementing a mindfulness program.

Our results showed that only one intervention group (classic ER-patient-centered AD) affected any mediators compared to the control condition. This could be due to the need to tailor each message to different patients or subgroups of patients with cLBP. Some patients may respond better to physician recommendations and scientific evidence while others prefer testimonials from similar patients. Some patients with cLBP may need flexible mindfulness training to fit their busy lives while others need externally imposed structure to be successful. The possible influence of the COVID-19 pandemic could also explain our general lack of intervention effects. The COVID-19 pandemic led to a rapid implementation of telehealth and teleconferencing. Some participants were recruited during the first months of the pandemic and this could have substantially increased acceptance of and comfort with mindfulness telehealth options as well as influenced participants to prefer flexible telehealth options to avoid COVID-19 infection. The COVID-19 pandemic could have also had the opposite effect for some participants, making them more likely to prefer longer in-person classes because they were experiencing less social contact due to the pandemic. Potential effects of the pandemic could have made intervention effects harder to detect. Regardless, any potential effects of the pandemic further emphasize the need to tailor messaging and likely treatment to each patient and also to the greater social context.

Previous research has also suggested that mindfulness training may need to be tailored to different patient populations. A meta-analysis of mindfulness training for chronic low back pain found that studies of older adults reduced the length of the sessions (1.5 h instead of 2.5) and a study of opioid users also adapted mindfulness training with cognitive behavioral therapy [22]. The adaptations from these previous studies are consistent with our findings suggesting the need to tailor mindfulness training to each patient. Tailoring mindfulness training for people using opioids for chronic low back pain treatment may warrant special consideration. Although mindfulness training is recommended as a treatment before medications, patients may be concerned about mindfulness training potentially replacing medications. Opioids in particular may cause fogginess and interfere with focus during physical meditations. When implementing mindfulness training, the intervention may need to be tailored to individual patients based on demographic factors such as age but based on the other treatments a patient currently uses for chronic low back pain.

The study results have several implications for clinical practice. For healthcare systems planning to implement mindfulness training for chronic low back pain, offering options to complete classes may be key. Some patients may prefer structured, in-person training while others need more flexible, drop-in arrangements. Some patients may need online classes instead of in-person training. Some patients may respond better to structured programs while others want to try different mindfulness techniques based on their own preferences. Messages promoting mindfulness training may need to be varied between scientific evidence and patient testimonials to improve acceptability. However, a mindfulness training program that is adaptable to each patient can also be the most resource- and time-intensive intervention to implement. The need for more resources and time can also challenge the sustainability of such a mindfulness training program. Future studies would be needed to investigate the best strategies for tailoring the delivery of mindfulness training to maximize adoption as well as how to balance sustainability with a flexible intervention.

The limitations of this study should be emphasized. Our outcome measure was self-reported intentions and not actual participation in mindfulness training. The study design was not longitudinal so the durability of the effects on intentions cannot be established from these data. Participants also came from Washington State and results might not generalize to other parts of the USA or other countries. The study strengths of using an experimental design, testing multiple mediators, and using patient feedback help balance these limitations.

## Conclusions

More research is needed on the dissemination of mindfulness training to help ensure this effective treatment reaches patients with chronic low back pain. Both our study results and previous research on mindfulness training for chronic low back pain support the importance of tailoring messaging about mindfulness to specific patient subgroups or to each individual patient. The only message that consistently influenced mediators was the version with scientific evidence (classic) and a highly flexible program (patient-centered). Research on strategies for tailoring education about mindfulness training could help address which message to use with which patient and when. As this study did not test tailoring of the mindfulness program, additional work is needed on tailoring mindfulness training in addition to messaging. Future studies should also use participation in mindfulness training as an outcome in addition to intentions. Mindfulness training is an effective treatment for chronic low back pain, and research to improve adoption of mindfulness treatment could help address this large public health concern.

## Supplementary Information

**Additional file 1:** All classic.

**Additional file 2:** All patient.

**Additional file 3:** Classic ER patient.

**Additional file 4:** Patient ER classic.

## Data Availability

A de-identified dataset is available upon reasonable request.
