# Supplementary material for: An experimental study to inform adoption of mindfulness-based stress reduction in chronic low back pain
Source: Implement Sci Commun. 2022 Aug 6;3:87. doi: 10.1186/s43058-022-00335-w (PMC9356436; doi:10.1186/s43058-022-00335-w)
Supplement: Supplementary file 1 — Additional file 1: All classic. [file 43058_2022_335_MOESM1_ESM.docx]

**
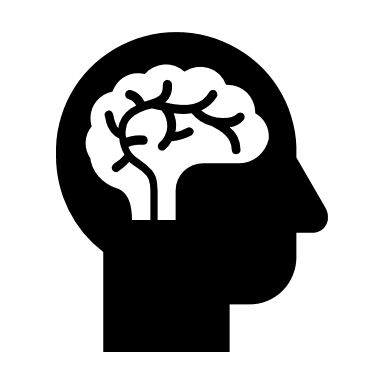
What is mindfulness?**

Mindfulness is a way of focusing your attention on the present moment. The idea is to be aware of your thoughts, feelings, and sensations without judging them. Mindfulness helps us change the way we relate to our thoughts, feelings, and physical sensations—including pain. The result is often that pain interferes less in our daily lives.

**
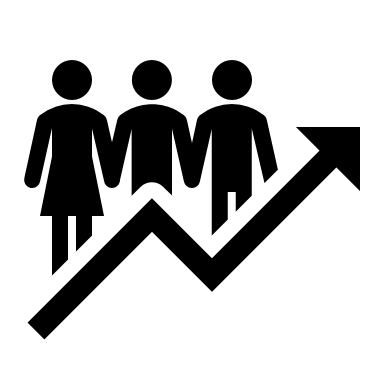
In fact, mindfulness is a scientifically proven treatment for chronic low back pain.** The American College of Physicians recommends mindfulness for chronic low back pain based on the results of several clinical research studies, one of which was done here at Kaiser Permanente Washington.

- These studies showed that mindfulness training led to benefits for people with chronic low back pain. These benefits included improvements in pain and the ability to engage in daily life activities.
- Benefits like these are similar to what people experience with other non-medication treatments, such as physical therapy.
- But more importantly, they are also similar to the benefits people experience from most pain medications—except without the side effects.

As you might know, opioids and other medications commonly prescribed for chronic pain can have severe side effects. To reduce these side effects, the Centers for Disease Control and Prevention now recommends non-medication treatments as the first line of therapy for chronic pain. For many people living with chronic pain, having fewer side effects from treatment helps them get back to living a fuller life and doing things they enjoy. Some people are also able to use fewer medications because of their mindfulness practice.

**
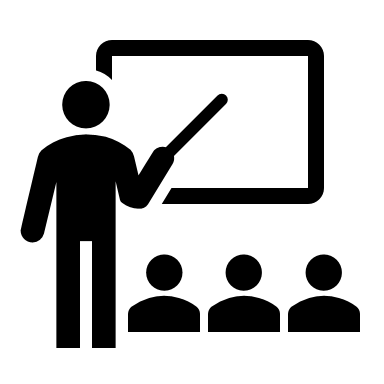
How do people learn mindfulness?**

Because mindfulness is a new experience for many people, the best way to learn it is by doing a mindfulness training course. Mindfulness training was developed in the 1970s by Dr. Jon Kabat-Zinn at the University of Massachusetts Medical Center to teach people a secular form of mindfulness.

- Mindfulness training is an in-depth course that you attend in person so that you can practice your skills.
- The course is taught in a group setting and led by a trained instructor.
- Classes are held once a week for 8 weeks. Each class lasts about 2 to 2.5 hours.
- Each week, the course will cover 1 or 2 mindfulness techniques that help people learn how to pay attention in a natural way.

The course starts with the simple act of eating a raisin slowly and with great awareness. Then it moves on to many other exercises that help people live with awareness and attention in the present moment. Over time, people improve their skills and have a deeper experience of mindfulness.

**
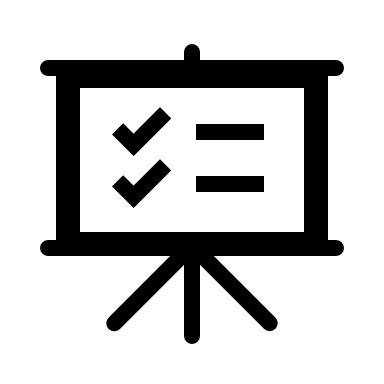
Mindfulness training teaches 3 core techniques:**

1. **The Body Scan** teaches you to use your breath to focus your attention on different parts of your body, one at a time. This helps you become more aware of your body’s sensations and the thoughts behind them.
2. **Mindful Movement** focuses on being aware of your breath from moment to moment as you move slowly through a series of gentle postures. This is different than other types of movement that focus on holding a challenging pose or posture. Being aware of the sensations in your body is the key point of this technique.
3. **Meditation** involves paying attention and focusing your mind while being still for an extended period of time. You can meditate while sitting or lying down. Meditation is about simply noticing your physical sensations, thoughts, and feelings without any judgement.

Mindfulness training will also teach you other techniques such as Walking Meditation and Mindful Eating.

**Mindfulness training involves many steps:**

- Introducing you to the concept of mindfulness
- Learning the three core techniques in a mindful way
- Practicing each technique at home

**Mindfulness is a complex skill:**

- As with any skill, people need to practice to become comfortable with it.
- When doing mindfulness training, you should practice for about 20 to 45 at home each day. This will help you build your mindfulness skills and use them in your daily life.
- The course will provide a book, handouts, and audio recordings to help guide your practice at home.

**
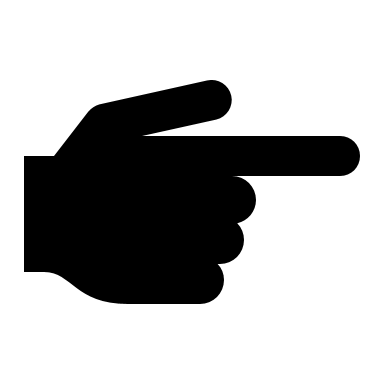
**

**Summary**

Mindfulness training is a structured program that will teach you the skill of mindfulness. Learning mindfulness will help change the way you experience pain and other sensations, which can improve your quality of life.
