# Supplementary material for: An experimental study to inform adoption of mindfulness-based stress reduction in chronic low back pain
Source: Implement Sci Commun. 2022 Aug 6;3:87. doi: 10.1186/s43058-022-00335-w (PMC9356436; doi:10.1186/s43058-022-00335-w)
Supplement: Supplementary file 2 — Additional file 2: All patient. [file 43058_2022_335_MOESM2_ESM.docx]

**
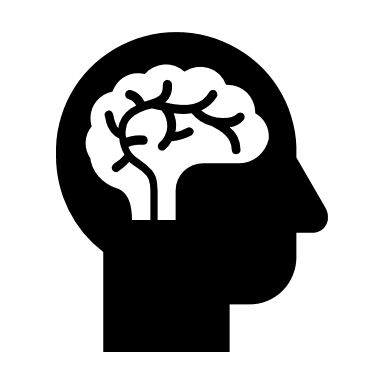
What is mindfulness?**

Mindfulness is a way of focusing your attention on the present moment. The idea is to be aware of your thoughts, feelings, and sensations without judging them. Mindfulness helps us change the way we relate to our thoughts, feelings, and physical sensations—including pain. The result is often that pain interferes less in our daily lives.

**In fact, mindfulness is a helpful treatment for chronic low back pain.** People around the world have used mindfulness to help with chronic low back pain because they experience positive results.

**
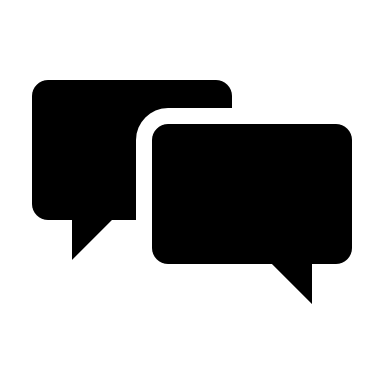
**

**Here are some comments from people who have used mindfulness to improve their pain or make it less disruptive in their lives:**

“I was very pleased to learn that there is something I can do when I am feeling pain. This is a definite improvement over feeling helpless. Mindfulness gives me confidence that I can really help myself, which is a wonderful feeling. There are many techniques I can use to help me to be aware in the present moment. I can also look at other things that are going on in my life, like how I interact with people, and make changes so that I am happier in my relationships. I have learned to pause and delay my reactions, which has been very helpful for me. When I connected with others in my mindfulness class, I learned that I am not alone in my pain.”

“Before I took a mindfulness class, I was in pain all the time. I couldn’t even walk a block without pain. The pain affected my mood and how I interacted with people. But things are different for me now. I can walk again without pain. And learning mindfulness has had a stronger effect for me as time goes on. I took the class about a year ago, and I have been practicing consistently ever since. I think this benefit comes from the fact that I have been so consistent in my practice. There are many ways to practice mindfulness, and I especially like the awareness exercises, the meditation, and the breathing. I now do stretching and meditation almost every night, at least 5 nights a week. And as long as I am not under a lot of stress, my pain is minimal. I no longer need to take medication. I feel like I have learned how to deal with the pain, rather than hide from it.”

**
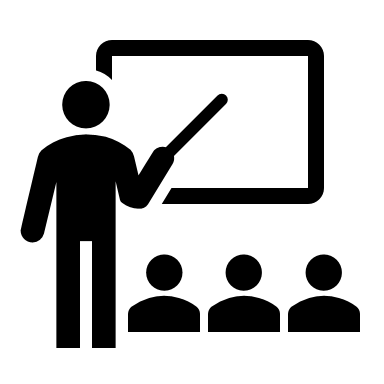
How do people learn mindfulness?**

Mindfulness is a natural way of paying attention. Mindfulness training will introduce you to a variety of techniques that help you harness your natural skills. You can try each technique and continue using those you find helpful.

Getting started with mindfulness training is easy. The class begins with an in-person orientation session so you can learn more about mindfulness before starting the training. You will also get an overview of the course and what the different sessions will cover. This will help you decide if the course is a good fit for you and to choose the sessions that are most interesting to you.

Classes are held weekly for 5-8 weeks—or longer if more classes would be helpful for you. Depending on what works best for you, classes last for 1 or 2 hours. You can either attend them in-person at a Kaiser Permanente facility or view them online in the privacy of your own home. Classes are recorded in case you miss one or want to view a specific class again. It’s fine to do a combination of in-person and online classes, if that works best for you.

**
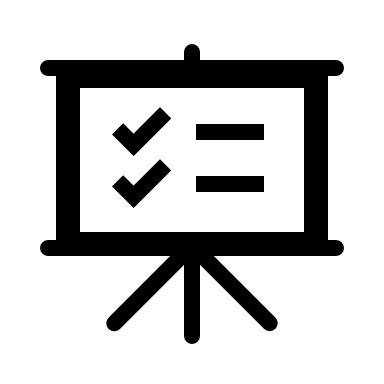
Mindfulness training will teach you 3 core techniques that you can continue to practice at home:**

1. **The Body Scan** teaches you to use your breath to focus your attention on different parts of your body, one at a time. This helps you become more aware of your body’s sensations and the thoughts behind them.
2. **Mindful Movement** focuses on being aware of your breath from moment to moment as you move slowly through a series of gentle postures. This is different than other types of movement that focus on holding a challenging pose or posture. Being aware of the sensations in your body is the key point of this technique.
3. **Meditation** involves paying attention and focusing your mind while being still for an extended period of time. You can meditate while sitting or lying down. Meditation is about simply noticing your physical sensations, thoughts, and feelings without any judgement.

Mindfulness training also offers some optional techniques such as Walking Meditation and Mindful Eating. Although consistent practice at home is recommended, finding a schedule for home practice that works for you is most important. Practicing at home helps you become comfortable with the skills and to use the techniques you find most helpful. The class provides audio tools to assist with your practice. You can even add the practice of mindfulness while doing some of your other usual activities, such as exercising.

**
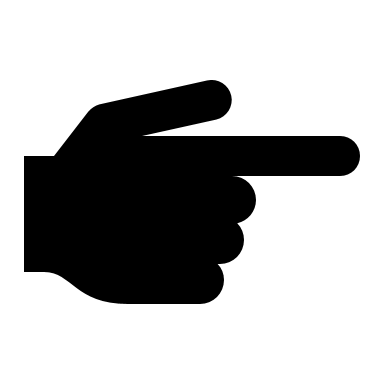
**

**Summary**

Mindfulness training is a flexible program designed to help you harness your natural ability to be mindful. This will help you change the way you experience pain and other sensations, which can improve your quality of life.
