# Supplementary material for: An experimental study to inform adoption of mindfulness-based stress reduction in chronic low back pain
Source: Implement Sci Commun. 2022 Aug 6;3:87. doi: 10.1186/s43058-022-00335-w (PMC9356436; doi:10.1186/s43058-022-00335-w)
Supplement: Supplementary file 3 — Additional file 3: Classic ER patient. [file 43058_2022_335_MOESM3_ESM.docx]

**
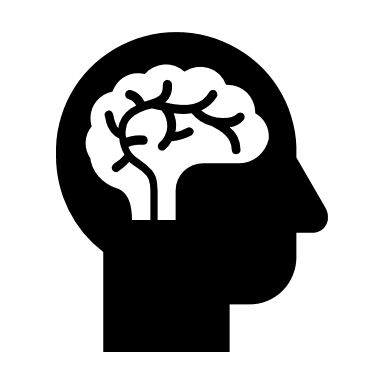
What is mindfulness?**

Mindfulness is a way of focusing your attention on the present moment. The idea is to be aware of your thoughts, feelings, and sensations without judging them. Mindfulness helps us change the way we relate to our thoughts, feelings, and physical sensations—including pain. The result is often that pain interferes less in our daily lives.

**
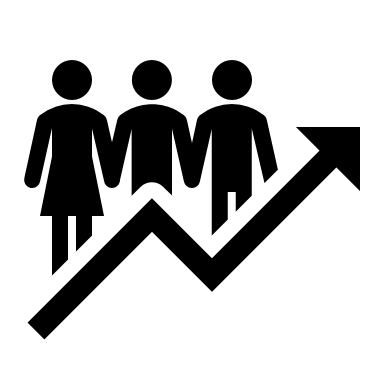
In fact, mindfulness is a scientifically proven treatment for chronic low back pain.** The American College of Physicians recommends mindfulness for chronic low back pain based on the results of several clinical research studies, one of which was done here at Kaiser Permanente Washington.

- These studies showed that mindfulness training led to benefits for people with chronic low back pain. These benefits included improvements in pain and the ability to engage in daily life activities.
- Benefits like these are similar to what people experience with other non-medication treatments, such as physical therapy.
- But more importantly, they are also similar to the benefits people experience from most pain medications—except without the side effects.

As you might know, opioids and other medications commonly prescribed for chronic pain can have severe side effects. To reduce these side effects, the Centers for Disease Control and Prevention now recommends non-medication treatments as the first line of therapy for chronic pain. For many people living with chronic pain, having fewer side effects from treatment helps them get back to living a fuller life and doing things they enjoy. Some people are also able to use fewer medications because of their mindfulness practice.

**
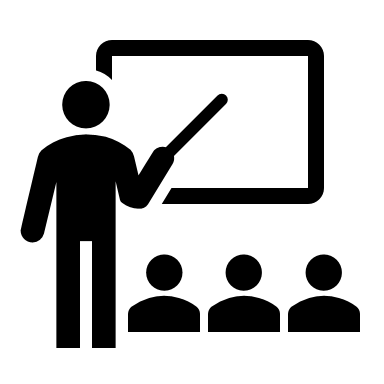
How do people learn mindfulness?**

Mindfulness is a natural way of paying attention. Mindfulness training will introduce you to a variety of techniques that help you harness your natural skills. You can try each technique and continue using those you find helpful.

Getting started with mindfulness training is easy. The class begins with an in-person orientation session so you can learn more about mindfulness before starting the training. You will also get an overview of the course and what the different sessions will cover. This will help you decide if the course is a good fit for you and to choose the sessions that are most interesting to you.

Classes are held weekly for 5-8 weeks—or longer if more classes would be helpful for you. Depending on what works best for you, classes last for 1 or 2 hours. You can either attend them in-person at a Kaiser Permanente facility or view them online in the privacy of your own home. Classes are recorded in case you miss one or want to view a specific class again. It’s fine to do a combination of in-person and online classes, if that works best for you.

**
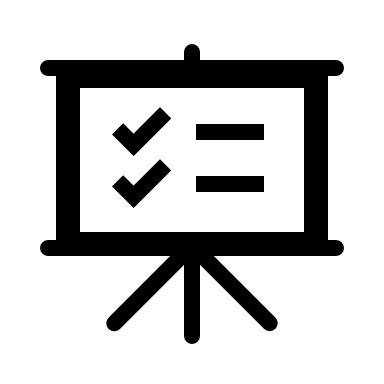
Mindfulness training will teach you 3 core techniques that you can continue to practice at home:**

1. **The Body Scan** teaches you to use your breath to focus your attention on different parts of your body, one at a time. This helps you become more aware of your body’s sensations and the thoughts behind them.
2. **Mindful Movement** focuses on being aware of your breath from moment to moment as you move slowly through a series of gentle postures. This is different than other types of movement that focus on holding a challenging pose or posture. Being aware of the sensations in your body is the key point of this technique.
3. **Meditation** involves paying attention and focusing your mind while being still for an extended period of time. You can meditate while sitting or lying down. Meditation is about simply noticing your physical sensations, thoughts, and feelings without any judgement.

Mindfulness training also offers some optional techniques such as Walking Meditation and Mindful Eating. Although consistent practice at home is recommended, finding a schedule for home practice that works for you is most important. Practicing at home helps you become comfortable with the skills and to use the techniques you find most helpful. The class provides audio tools to assist with your practice. You can even add the practice of mindfulness while doing some of your other usual activities, such as exercising.

**
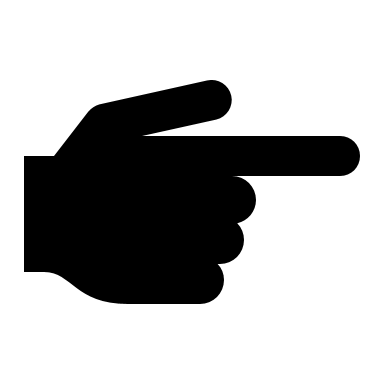
**

**Summary**

Mindfulness training is a flexible program designed to help you harness your natural ability to be mindful. This will help you change the way you experience pain and other sensations, which can improve your quality of life.
