# Supplementary material for: An experimental study to inform adoption of mindfulness-based stress reduction in chronic low back pain
Source: Implement Sci Commun. 2022 Aug 6;3:87. doi: 10.1186/s43058-022-00335-w (PMC9356436; doi:10.1186/s43058-022-00335-w)
Supplement: Supplementary file 4 — Additional file 4: Patient ER classic. [file 43058_2022_335_MOESM4_ESM.docx]

**
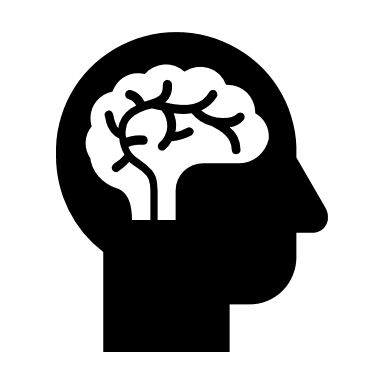
What is mindfulness?**

Mindfulness is a way of focusing your attention on the present moment. The idea is to be aware of your thoughts, feelings, and sensations without judging them. Mindfulness helps us change the way we relate to our thoughts, feelings, and physical sensations—including pain. The result is often that pain interferes less in our daily lives.

**In fact, mindfulness is a helpful treatment for chronic low back pain.** People around the world have used mindfulness to help with chronic low back pain because they experience positive results.

**
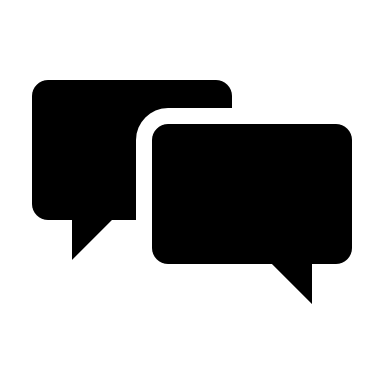
**

**Here are some comments from people who have used mindfulness to improve their pain or make it less disruptive in their lives:**

“I was very pleased to learn that there is something I can do when I am feeling pain. This is a definite improvement over feeling helpless. Mindfulness gives me confidence that I can really help myself, which is a wonderful feeling. There are many techniques I can use to help me to be aware in the present moment. I can also look at other things that are going on in my life, like how I interact with people, and make changes so that I am happier in my relationships. I have learned to pause and delay my reactions, which has been very helpful for me. When I connected with others in my mindfulness class, I learned that I am not alone in my pain.”

“Before I took a mindfulness class, I was in pain all the time. I couldn’t even walk a block without pain. The pain affected my mood and how I interacted with people. But things are different for me now. I can walk again without pain. And learning mindfulness has had a stronger effect for me as time goes on. I took the class about a year ago, and I have been practicing consistently ever since. I think this benefit comes from the fact that I have been so consistent in my practice. There are many ways to practice mindfulness, and I especially like the awareness exercises, the meditation, and the breathing. I now do stretching and meditation almost every night, at least 5 nights a week. And as long as I am not under a lot of stress, my pain is minimal. I no longer need to take medication. I feel like I have learned how to deal with the pain, rather than hide from it.”

**
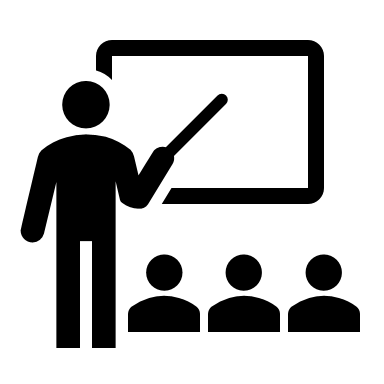
How do people learn mindfulness?**

Because mindfulness is a new experience for many people, the best way to learn it is by doing a mindfulness training course. Mindfulness training was developed in the 1970s by Dr. Jon Kabat-Zinn at the University of Massachusetts Medical Center to teach people a secular form of mindfulness.

- Mindfulness training is an in-depth course that you attend in person so that you can practice your skills.
- The course is taught in a group setting and led by a trained instructor.
- Classes are held once a week for 8 weeks. Each class lasts about 2 to 2.5 hours.
- Each week, the course will cover 1 or 2 mindfulness techniques that help people learn how to pay attention in a natural way.

The course starts with the simple act of eating a raisin slowly and with great awareness. Then it moves on to many other exercises that help people live with awareness and attention in the present moment. Over time, people improve their skills and have a deeper experience of mindfulness.

**
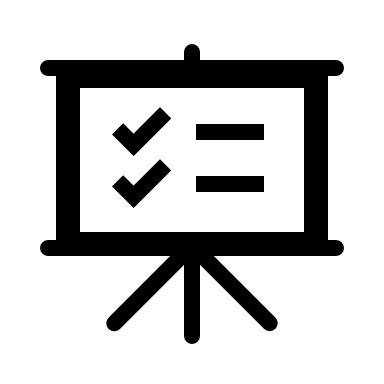
Mindfulness training teaches 3 core techniques:**

1. **The Body Scan** teaches you to use your breath to focus your attention on different parts of your body, one at a time. This helps you become more aware of your body’s sensations and the thoughts behind them.
2. **Mindful Movement** focuses on being aware of your breath from moment to moment as you move slowly through a series of gentle postures. This is different than other types of movement that focus on holding a challenging pose or posture. Being aware of the sensations in your body is the key point of this technique.
3. **Meditation** involves paying attention and focusing your mind while being still for an extended period of time. You can meditate while sitting or lying down. Meditation is about simply noticing your physical sensations, thoughts, and feelings without any judgement.

Mindfulness training will also teach you other techniques such as Walking Meditation and Mindful Eating.

**Mindfulness training involves many steps:**

- Introducing you to the concept of mindfulness
- Learning the three core techniques in a mindful way
- Practicing each technique at home

**Mindfulness is a complex skill:**

- As with any skill, people need to practice to become comfortable with it.
- When doing mindfulness training, you should practice for about 20 to 45 minutes at home each day. This will help you build your mindfulness skills and use them in your daily life.
- The course will provide a book, handouts, and audio recordings to help guide your practice at home.

**
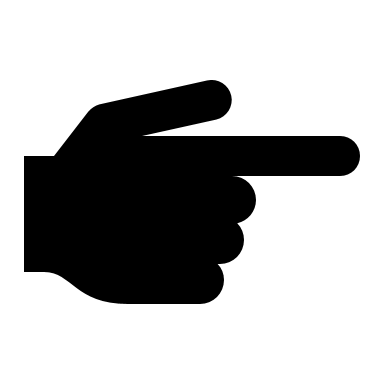
**

**Summary**

Mindfulness training is a structured program that will teach you the skill of mindfulness. Learning mindfulness will help change the way you experience pain and other sensations, which can improve your quality of life.
